# Institutional Open Access Funds: Now Is the Time

**DOI:** 10.1371/journal.pbio.1000375

**Published:** 2010-05-25

**Authors:** Charles D. Eckman, Beth T. Weil

**Affiliations:** Library, University of California Berkeley, Berkeley, California, United States of America

The Great Recession is making the hard writing on the wall for research libraries easy to read. In the United States, drastic decreases in endowment income at private universities have been well-publicized. Most public universities and research institutions that rely upon public funding are now experiencing reductions of a similar scale [1]. As university income has declined, reductions have been assigned to library collections funds [2]. This has a downstream effect on the scholarly society and commercial publishers who rely upon institutional subscriptions and licenses for revenue. Statements have been issued by library coalitions pleading for journal publishers to respond by issuing price reductions [3],[4]. Some publishers have responded by keeping journal prices flat. However, the signs are clear: more and more publishers are likely to find themselves challenged to survive through maintaining the still dominant funding model. That model is characterized by institutional subscriptions to a set of articles tied to a single journal's brand or an entire publisher's brand (in the case of the so-called “big deal”) providing the institution's researchers with entrée to the content behind walls.

As many, if not all, academic libraries closely evaluate journal- and publisher-based subscription content, broad research access to journal literature will fall as libraries cancel those subscriptions and licenses. As a result, researchers will increasingly find themselves reliant upon informal exchange networks (for closed-access content) and open-access (OA) repositories (for open-access content) for obtaining access to research findings [5]. Researchers choosing a publication venue based on assumptions about a journal or publisher whose brand has traditionally been widely subscribed may be disappointed to learn that a growing number of their peers will not have access to the content. Other factors, such as access to the journal article, should and will increasingly be viewed as a principal factor in their choice of publication venue. *Now* is the time for research institutions (including libraries) to establish new fund flows in support of open-access publishing.

## Publishing Options and Fund Flows

Open access means that publishers make research available to readers at no cost immediately upon publication and place no restrictions on use with attribution. Sustaining “pure OA” publishing, in which all the articles included in a journal issue are OA. requires a revenue stream. The business models for supporting open access are varied. These include, in various combinations, community support, advertising, sponsorship, institutional support, hard copy support, article processing charges, institutional membership, and collaborative purchasing [6]. Within the set of high-impact OA journals, the publishing fee model is predominant; individual charges range from US$530 to US$5,000 [7],[8].

Subscription publishers are aware of the inherent attractiveness to both authors and readers of open access as a dissemination mechanism. This explains the recent, dramatic emergence of the “hybrid” OA journal phenomenon, with several publishers beginning to offer OA on an article-by-article basis. An author charge allows them to do this, and the charges range widely from under US$100 to US$5,000 in the case of Nature Publishing Group [9]. As some of these hybrid options have met with sufficient success, some journal publishers are reducing subscription costs based on the offsets provided by these author-processing charges.

There have been significant and positive changes in the prospects for changing funding streams to support open-access publishing since Stuart Shieber, Director of Harvard's Office for Scholarly Communication, argued for funding equity in an earlier issue of *PLoS Biology*. Much attention has surrounded the Compact for Open Access Publishing Equity (COPE) (http://www.cope.org), reflecting the explicit commitment of its members to establish “durable mechanisms for underwriting reasonable publication charges for articles written by its faculty and published in fee-based open-access journals and for which other institutions would not be expected to provide funds.” Lest there be any confusion that this recommendation is limited to the eight COPE institutions, in the last year the number of universities and research institutions worldwide with an open-access publishing fund has grown dramatically. Within North America the number of institutions with open-access funds has grown from two to 15 within a two-year period. [10] A growing number of research-sponsoring groups, including governmental agencies and research funders, have established funds to support open access publishing [11].

Some observers strongly argue that the time to establish an OA fund is only after a Green OA mandate has been established. However, 13 out of 15 central funds in North America have been established absent any clear institution-wide Green OA mandates. The value of establishing these funds independent of an institution consensus on Green OA mandate is being demonstrated in practice.

It might be argued that the current economic climate is not conducive to the establishment of such a fund. As noted earlier, libraries have been hit particularly hard during the current economic crisis. In fact, the need to dramatically reduce expenditures on information resources offers an opportunity to expand barrier-free access for readers to the results of academic and scientific inquiry. The strategic redirection of a small percentage of the funds that a large number of research-intensive institutions are currently investing in subscription-based publishing toward OA publication fees could both reduce overall costs and dramatically expand access to research. The experiences of those institutions that have established such funds indicate that the overhead involved is manageable and sustainable. This article is informed by the authors' experience at Berkeley in establishing one of the first such funds, but draws upon knowledge of other fund experiences. A complete guide to setting up an OA fund—along with detailed information regarding the operation of existing funds—is available on a new ARL-SPARC resource [12].

## Berkeley Research Impact Initiative

Reflecting the institution's interest in broad dissemination of its research findings and a sustainable scholarly communications environment, the Berkeley Research Impact Initiative (BRII) was unveiled in January 2008 as an 18-month pilot project endorsed jointly by the University Librarian and Vice Chancellor for Research. Formal project goals include: increase the amount of Berkeley research accessible to readers; promote faculty rights retention in their journal publishing output; provide Berkeley researchers with funds to encourage the use of new and innovative scholarly publishing outlets; support researchers who would like to publish open-access articles but for whom cost is a barrier; encourage a more sustainable scholarly communication environment; and develop infrastructure for supporting alternatives to subscription-based publishing.

Library collections budgets are allocated to cover a range of scholarly communication costs, including book and journal acquisitions (binding, cataloging services, memberships that bring in content, etc.). During the emergence of digital journal and database options in the 1990s, a significant portion of the budget was directed in part toward licensing these new online resources. Now, these budgets can and should support investments in the open-access publishing choices of researchers, in keeping with the public dissemination research mission of the university. Investing collection dollars in this way manages the transition to a more sustainable model of publishing, ensuring that the library serves as the integrating agency (as the one point within the research university with a broad perspective on its publishing activities and how they relate to the scholarly communications environment).

The BRII covers publication charges for open-access publishers such as PLoS, BioMedCentral, PhysMathCentral, and others (capped at US$3,000). It also covers a maximum of US$1,500 of publication charges for hybrid journals that offer authors the option to make their article free-to-read and provide for non-commercial re-use with attribution (“libre OA”) immediately upon publication—authors are responsible for any hybrid charges that exceed US$1,500. The notion of hybrid support emerged during stakeholder negotiations. We made a strategic choice to fund both OA and limited hybrid charges because including hybrid charges ensured administrative and academic approval of the initiative.

Allowing hybrid support has increased the number of articles eligible for funding and has thus allowed more faculty to engage in a form of OA publishing. Broadening the pool of potential applicants and publishers has also generated numerous conversations about copyright, open access, and scholarly communication in general among faculty, librarians, and publishers—conversations that are directly attributable to this component of the program.

The principal objection to supporting hybrid-access articles is that this encourages publishers to engage in “double-dipping,” by charging both subscription fees and author publication fees). However, we know that our faculty takes advantage of these options independently of our OA fund. Controlling costs in such a context presents seemingly insurmountable barriers because, as Shieber has pointed out, “publishers practice price discrimination, bundling, and price changes over time, which separately and together make it impossible to tell what a subscriber's costs would have been absent the hybrid fee discount” [13]. Without initiatives that track this fund flow on campus, we have very little understanding of the character and scope of the phenomenon. Including limited hybrid support in an OA fund is one way to help us understand the full scope of that activity at Berkeley. We cannot realistically make effective claims on publishers to reduce our institution's subscription charges to their journals in light of our institution's expenditures on paid access fees for particular articles unless we as an institution can track them. This component of the fund strengthens our stewardship role and data-gathering abilities regarding the institution's investment in research dissemination and access. Calgary, to a great extent, and Berkeley (to a lesser extent) go back to the publishers when we can to seek some form of reduced subscription fees in acknowledgment of our paid access charges.

Whether or not we are able to convince publishers at the local level to track and reduce our subscription charges, we know that some publishers are reducing overall subscription charges in relationship to the uptake of the paid access, hybrid option. Perhaps the most consistent position in this regard has been that of Oxford University Press, which has for three straight years incorporated OA uptake into its subscription pricing decisions, as well as the American Physiological Society [14],[15]. We believe with others that hybrid publishers should commit publicly to reducing their subscription charges in direct proportion to the uptake of their article OA charges.

Including a modest hybrid subsidy within an OA fund is not an end-game, but a transitional strategy that will add both knowledge and power to institutions in their negotiations with publishers at the same time that it encourages OA experimentation among authors and publishers. Institutions are likely to include hybrid support in their OA funds as publishers become more transparent about their OA revenue. Publishers can support transparency by offering lower publishing fees to authors at institutions with subscriptions and regular reporting on OA uptake by authors to institutions. By providing a low cap on reimbursements for hybrid OA options, institutions introduce a measure of cost control and initiate conversations with authors on the value of OA for overcoming the dissemination challenge posed by the declining library subscription base.

## Fostering Dialogue

The discussions that took place on the Berkeley campus prior to the establishment of the OA Fund were critical ones. These included conversations with the Senate Committee on the Library representing a diversity of disciplines and perspectives on the state of scholarly communication. The scope and character of the program was shaped by these discussions.

In addition to the stewardship value, we have discovered that the interaction with researchers when we discussed the differential caps for pure OA and hybrid OA provides an ideal opportunity to face the economic choices and copyright implications. We find ourselves in a unique position to describe one-on-one with authors the scale of library investments in subscription content. The purpose is not to change their choice of publisher, but rather to increase their awareness of options and implications for access and sustainable budgeting in the long term. Fostering this awareness is critical since part of the dysfunction of the current scholarly communication landscape is that the authors are shielded (through the institutional subscription model) from the economic impact of their microeconomic choices.

The dialogue is not limited to intra-institutional contexts. In several situations, the researcher or library fund coordinator found him/herself in a conversation with publishers regarding whether the pure or hybrid funding cap applied and whether a subscription or membership-based discount would apply. Again, this fosters increased dialogue as well as better awareness of the actual fund flows between the institution, author, and the publisher. Through this process, a richer picture of the full scope of funding available to researchers for publishing has been revealed. For example, we learned by reviewing reports from PLoS and BMC that over 75% of Berkeley researchers who chose to publish in one of these two OA venues had the funding to do this without requiring Berkeley institutional funds. Assuming that Berkeley is not atypical in this regard, this suggests that it is financially feasible for research-intensive universities to invest in an OA fund for the small percentage of researchers who do not have the means to fund their OA publishing.

The fund has also fostered significant dialogue within the library and its constituents about the library's collections budget as an appropriate source of funding to sustain new models of scholarly communication, in the same fashion that we have in the past as formats and funding models have changed.

## Summary

To date, the Berkeley OA fund has ensured that 43 articles are free to be read immediately upon publication and 44 additional articles are now in the pipeline. The full scope of OA publishing during this period was significantly larger, at least by a factor of four. The OA fund allocation will continue to be carefully managed over the coming years. We are tracking our potential liability assuming the OA landscape grows with additional publishers and OA options. The amount predicted as necessary to maintain the fund based on the initial 18-month uptake data is US$45,000. This is less than 1% of the US$6.2 million the library invests in subscribing to closed-access digital journals. We are paying attention in particular to the attempts by the California Digital Library, which negotiates major journal publisher licenses on behalf of the UC campuses, to include terms within the licenses that enable UC authors to take advantage of publishers' hybrid OA options [16].

And it comes with far less of the myriad overhead costs associated with those closed-access subscriptions because that subscription price doesn't tell the full story of the actual cost of maintaining the subscription. Those subscriptions involve staff-intensive license negotiations. Institutions develop and maintain systems architectures in order to ensure that only authorized users have access and respond to challenges from publishers of the content when actual or potential breaches of the licenses are identified (publishers invest hugely in monitoring use of their content in order to ensure the license terms are not breached and are quite willing to contact the institutional subscriber when any untoward activity appears on their logs). They must, in certain instances, maintain the confidentiality of certain clauses in the licenses and increasingly respond to freedom of information, public records act requests related to the investments of public resources in those contracts.

The need to experiment is particularly heightened during this economic crisis when investments in subscriptions are increasingly difficult to justify, particularly given the alternate forms of open access to content and decreasing ability for libraries to reliably distinguish OA and non-OA content within the journal. We believe that institutions (and the sub-institutional units that manage collection funds) should be open to exploring alternative funding models for scholarly communication. Institutions should highly value funding models that promote universal access to their research output. And during an economic crisis, these institutions should question the extensive financial and human resource investments required by the subscription model, a model that both excludes non-authorized users and entails large-scale and complex licensing and legal obligations. The time is now for broad-scale adoption of institutional OA funds.
